# Population Genomic Scan for Candidate Signatures of Balancing Selection to Guide Antigen Characterization in Malaria Parasites

**DOI:** 10.1371/journal.pgen.1002992

**Published:** 2012-11-01

**Authors:** Alfred Amambua-Ngwa, Kevin K. A. Tetteh, Magnus Manske, Natalia Gomez-Escobar, Lindsay B. Stewart, M. Elizabeth Deerhake, Ian H. Cheeseman, Christopher I. Newbold, Anthony A. Holder, Ellen Knuepfer, Omar Janha, Muminatou Jallow, Susana Campino, Bronwyn MacInnis, Dominic P. Kwiatkowski, David J. Conway

**Affiliations:** 1 Medical Research Council Unit, Fajara, Banjul, The Gambia; 2 Department of Pathogen Molecular Biology, London School of Hygiene and Tropical Medicine, London, United Kingdom; 3 Wellcome Trust Sanger Institute, Hinxton, United Kingdom; 4 Weatherall Institute of Molecular Medicine, University of Oxford, Oxford, United Kingdom; 5 Division of Parasitology, MRC National Institute for Medical Research, London, United Kingdom; 6 Wellcome Trust Centre for Human Genetics, University of Oxford, Oxford, United Kingdom; Arizona State University, United States of America

## Abstract

Acquired immunity in vertebrates maintains polymorphisms in endemic pathogens, leading to identifiable signatures of balancing selection. To comprehensively survey for genes under such selection in the human malaria parasite *Plasmodium falciparum*, we generated paired-end short-read sequences of parasites in clinical isolates from an endemic Gambian population, which were mapped to the 3D7 strain reference genome to yield high-quality genome-wide coding sequence data for 65 isolates. A minority of genes did not map reliably, including the hypervariable *var*, *rifin*, and *stevor* families, but 5,056 genes (90.9% of all in the genome) had >70% sequence coverage with minimum read depth of 5 for at least 50 isolates, of which 2,853 genes contained 3 or more single nucleotide polymorphisms (SNPs) for analysis of polymorphic site frequency spectra. Against an overall background of negatively skewed frequencies, as expected from historical population expansion combined with purifying selection, the outlying minority of genes with signatures indicating exceptionally intermediate frequencies were identified. Comparing genes with different stage-specificity, such signatures were most common in those with peak expression at the merozoite stage that invades erythrocytes. Members of *clag*, *PfMC-2TM*, *surfin*, and *msp3*-like gene families were highly represented, the strongest signature being in the *msp3*-like gene PF10_0355. Analysis of *msp3*-like transcripts in 45 clinical and 11 laboratory adapted isolates grown to merozoite-containing schizont stages revealed surprisingly low expression of PF10_0355. In diverse clonal parasite lines the protein product was expressed in a minority of mature schizonts (<1% in most lines and ∼10% in clone HB3), and eight sub-clones of HB3 cultured separately had an intermediate spectrum of positive frequencies (0.9 to 7.5%), indicating phase variable expression of this polymorphic antigen. This and other identified targets of balancing selection are now prioritized for functional study.

## Author Summary

The memory component of acquired immune responses selects for distinctive patterns of polymorphism in genes encoding important target antigens of pathogens. These are detectable by surveying for evidence of balancing selection, as previously illustrated in analyses of genes encoding malaria parasite antigens that are candidate targets of naturally acquired immunity. For a comprehensive screen to discover targets of immunity in the major human malaria parasite *Plasmodium falciparum*, an endemic population in West Africa was sampled and genome sequence data obtained from 65 clinical isolates, allowing analysis of polymorphism in almost all protein-coding genes. Antigen genes previously studied by capillary re-sequencing in independent population samples had highly concordant indices in the genome-wide analysis here, and this has identified other genes with stronger evidence of balancing selection, now prioritized for functional study and potential vaccine candidacy. The statistical signatures consistent with such selection were particularly common in genes with peak expression at the stage that invades erythrocytes, and members of several gene families were represented. The strongest signature was in the *msp3*-like gene PF10_0355, so we studied the transcript and protein product in parasites, revealing an unexpected pattern of phase variable expression. Variation in expression of polymorphic antigens under balancing selection may be more common than previously thought, requiring further study to assess vaccine candidacy.

## Introduction

Evolutionary and population genetic analyses of pathogens should help discover mechanisms of pathogenesis, immune evasion and drug resistance. Application of these approaches to malaria parasites is a high priority, as there is an ongoing need to identify targets of immunity as potential vaccine candidates, and to understand and monitor the continuous evolution and emergence of drug resistance. Advances in genome sequencing methods now allow more comprehensive analyses of polymorphism within populations, and increases the efficiency of detecting signatures of natural selection from patterns of genetic variation [1], [2], [3], [4]. This also encourages the scaled up use of allele frequency-based methods for detection of recent and ongoing selection [5], [6].

*Plasmodium falciparum* causes more human disease than any other eukaryotic pathogen [7], and contains ∼5560 annotated genes in a compact genome of ∼23 megabase pairs (Mb) with a high recombination rate in each of 14 chromosomes [8], [9]. Previous analyses of microsatellites and single nucleotide polymorphisms (SNP) have identified selective sweeps around several previously-identified drug resistance genes, encouraging genome wide analyses to prospect for other chromosomal loci containing genes under recent positive directional selection [10], [11], [12]. Separately, studies of individual genes encoding surface-exposed protein targets of acquired immunity have shown signatures of balancing selection maintaining different alleles within populations (reviewed in [13]), and these results replicate well in independent studies of different endemic populations [13], [14], [15], [16], [17]. This indicates that new potential candidates for vaccine development based on multi-allelic antigen formulations might be identified with a systematic genome-wide scan for such signatures in an endemic population. The very low levels of linkage disequilibrium due to frequent recombination in highly endemic *P. falciparum* populations [10], [18], [19] means that contiguous sequence data are needed to allow an effective scan for signatures of balancing selection. The limited numbers and disparate sampling of *P. falciparum* genome sequences until recently have not enabled such frequency-based analyses to be effectively applied [11], [20], [21], [22]. More thorough analysis of sequence diversity within local populations is now possible by paired-end short-read sequencing of parasites in clinical isolates [23], [24], which facilitates new approaches.

Here, we present a genome-wide survey of polymorphism in coding sequences of *P. falciparum* in an endemic population sample of 65 Gambian clinical isolates, the largest sample of parasite genomes from a single location reported to date. We identified genes having polymorphic site frequency spectra consistent with effects of balancing selection, forming a prime catalogue of candidates for studies of immune mechanisms and potential vaccine development. Genes expressed at the merozoite stage were more likely than others to show such patterns, as were members of several small multigene families encoding surface and exported proteins that are yet to be studied intensively. The product of the gene with the strongest statistical signature was studied, revealing an unexpected pattern of variation in expression among different isolates and within individual parasite clones, suggesting that selection for phase variation may operate alongside selection for amino acid polymorphisms.

## Results

### Sequencing of an endemic population sample of malaria parasite isolates

**Figure 1:**
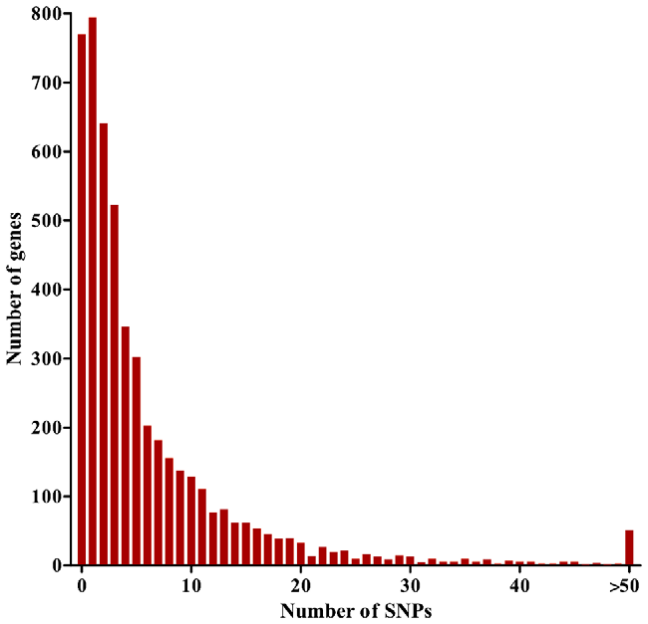
Distribution of numbers of SNPs per gene for 5,056 *P. falciparum* genes analyzed with a population sample of 65 Gambian clinical isolates.

We generated genome-wide short read sequences for each of 65 Gambian *P. falciparum* clinical isolates and aligned these to the 5560 gene coding sequences in the genome sequence of *P. falciparum* clone 3D7 (version 2.1), yielding sequence contigs for 5475 (98.5%) of the genes. The overall coding sequence coverage of each isolate was >80% (mean of 95.6%) at a read depth of 10 or more (Table S1). For each isolate, the consensus read sequence was taken as the majority parasite allele sequence for each gene. We excluded 419 genes from analysis that belonged to *var*, *rifin* or *stevor* hypervariable families, or that did not have more than 70% coverage at a read depth of 5 or more for at least 50 of the isolates, and thus proceeded to analyse sequences of 5056 genes (90.9% of all annotated in the genome). We identified 2203 genes with minimal or no polymorphism (769 with 0 SNPs, 794 with 1 SNP and 640 with 2 SNPs), and 2853 genes with at least 3 SNPs (mean coverage of the coding sequences of these genes was 98.5%). Genes with at least 3 SNPs were considered informative for comparisons of polymorphic nucleotide site frequency spectra in analyses that aimed to be as comprehensive as possible, although data for individual genes are inevitably statistically stronger for those with higher numbers of SNPs (1009 of these had ≥10 SNPs, and 51 had ≥50 SNPs; results for all individual genes are given in Table S2) (Figure 1).

### Polymorphic site frequency spectra in coding sequences

**Figure 2:**
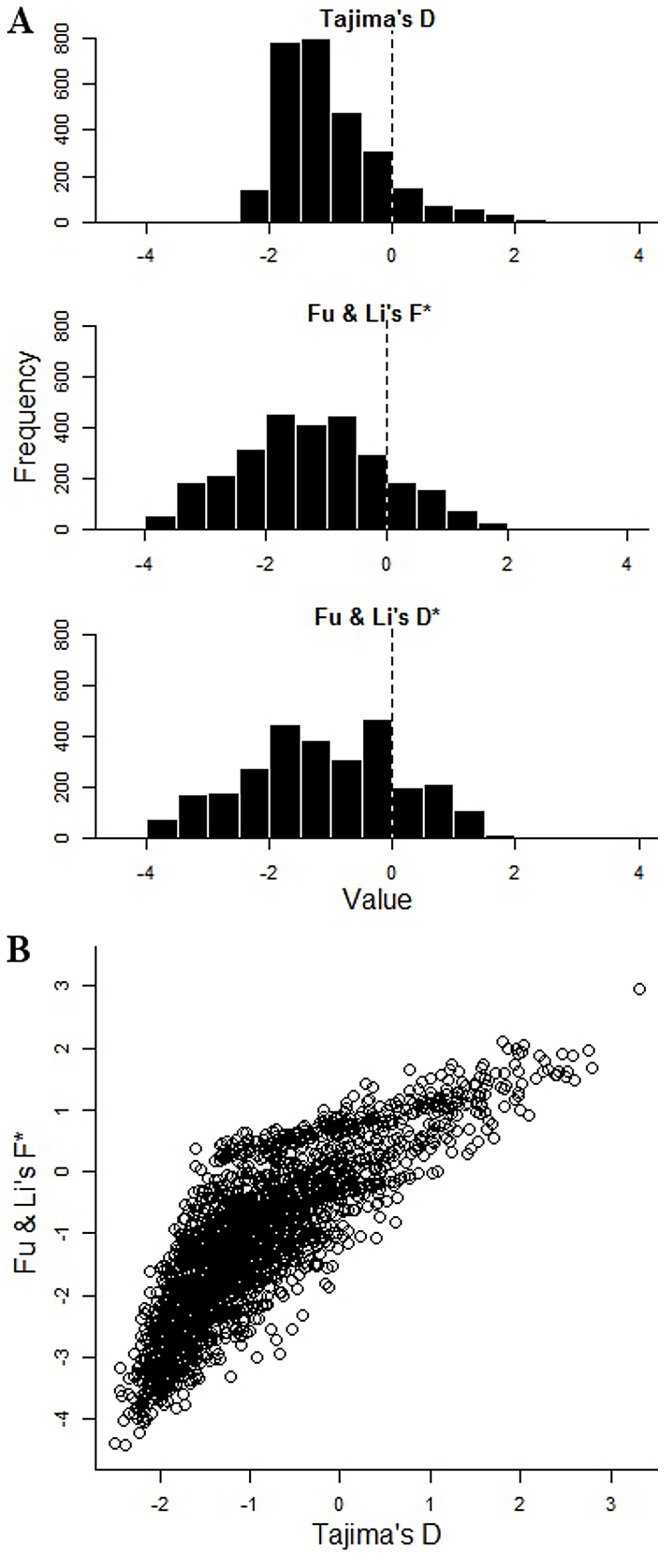
Tajima's and Fu & Li's summary indices of nucleotide site frequency spectrum for each of 2,853 *P. falciparum* genes with 3 or more SNPs in the Gambian population. A. Frequency distribution histograms for the individual gene values for Tajima's D, Fu & Li's F* and Fu & Li's D* respectively. B. Two-dimensional plot of Tajima's D and Fu & Li's F* values for each of the 2853 genes (*r* = 0.67; correlation between Fu & Li's F* and D* indices is stronger, *r* = 0.96; correlation between Tajima's D and Fu & Li's D* is less, *r* = 0.50; P<0.001 for all correlations). Those in the top right tail of the distribution with high indices of both are considered further as genes with candidate signatures of balancing selection.

Across all 2853 genes with 3 or more SNPs, values of Tajima's D and Fu & Li's indices were mostly negative (mean Tajima's D = −1.00, Fu & Li's D* = −1.14, F* = −1.24), indicating an excess of low frequency and singleton polymorphisms compared with that expected under neutrality for a population at mutation-drift equilibrium (Figure 2A). This is consistent with historical population expansion, as supported also by predominantly negative values of Fu's Fs index (mean = −7.1). As expected, values of Tajima D correlated with those of Fu & Li's F* (r = 0.68) (Figure 2B), and D* (r = 0.50), while Fu & Li's F* and D* indices were very highly correlated (r = 0.96).

**Figure 3:**
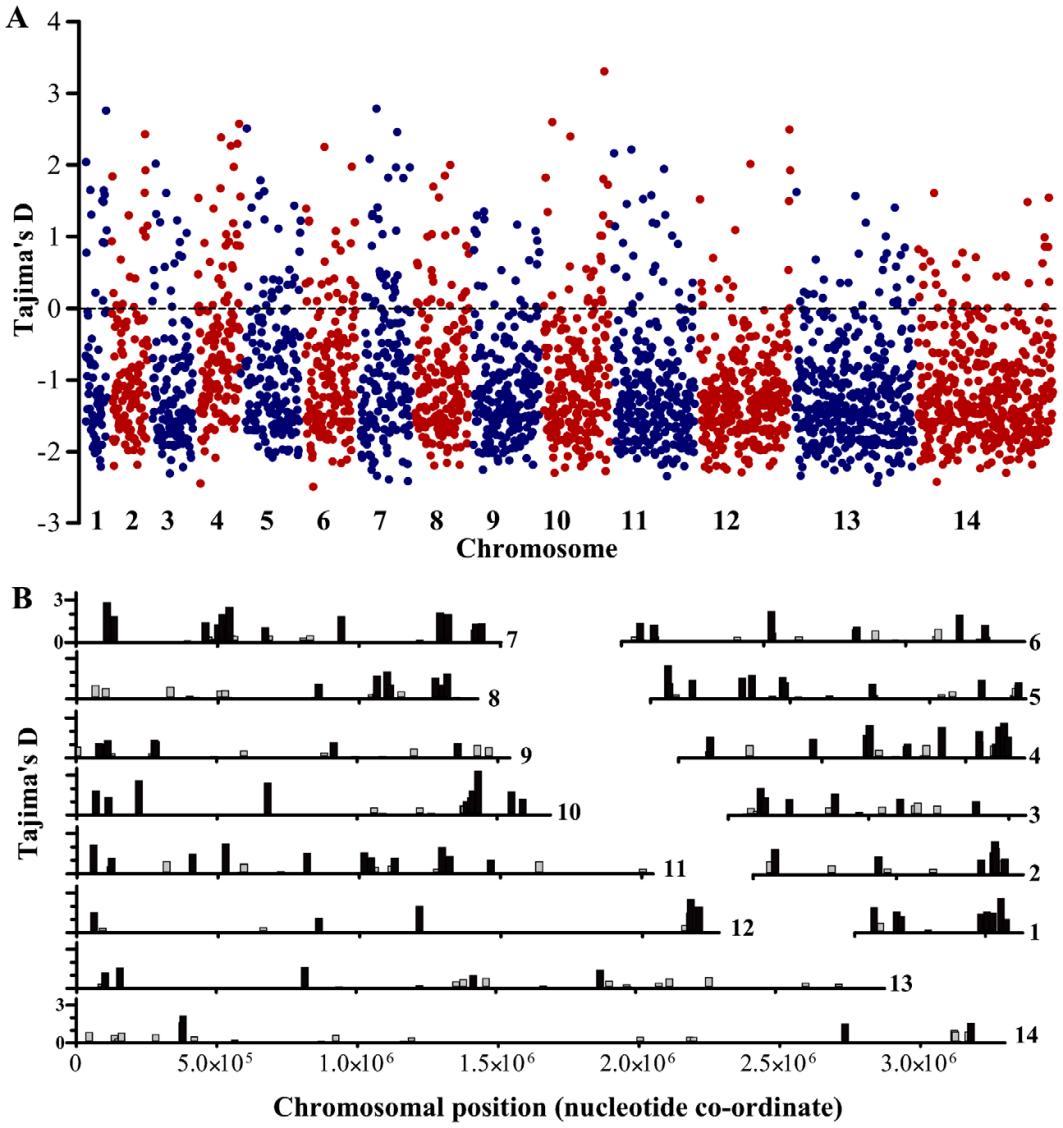
Distribution of Tajima's D values across all 14 chromosomes for each of the 2,853 *P. falciparum* genes with 3 or more SNPs in the Gambian population. A. All values for individual genes are plotted as individual points positioned according to the order of the genes along each of the chromosomes. B. Chromosomal locations of each of the genes with positive Tajima's D values (genes with values between zero and 1.0 are shown in grey, those with values >1.0 in black).

Genes with high Tajima's D values had a wide distribution across all chromosomes (Figure 3A). Overall, 337 (11.8%) of the 2853 genes with at least 3 SNPs had Tajima's D values above zero, of which 241 also had positive values for Fu & Li's F and D, and these loci were widely distributed throughout the genome (Figure 3B). Table 1 shows indices for the genes with the top 25 values of Tajima's D, among those having at least 10 SNPs. The full list of results for all of the 2853 genes with 3 or more SNPs is given in Table S2. Several of the genes at the top of the list encode antigens that are known targets of immunity, the most studied of which is the apical membrane antigen 1 (AMA1), against which many naturally-acquired and experimental vaccine-induced immune responses are allele-specific [25], [26], [27], [28]. The *ama1* gene previously showed very high Tajima's D values in independent studies from The Gambia [17] and other endemic populations [25], [29], [30], [31], and has long been recognised to have exceptional nucleotide diversity at nonsynonymous positions compared with synonymous positions [32], [33] as reflected also in the data here.

**Table 1:** 25 genes with ≥10 SNPs showing highest values of Tajima's D index in a genome-wide analysis of sequences of 65 Gambian *P. falciparum* isolates.

Gene ID	Chr	Product description	N	% CDS	S	π×10^−3^	dN/dS	Tajima's D	Fu & Li's D*	Fu & Li's F*	Peak Expression
PF10_0355	10	merozoite surface protein	1845	80	111	25.9	0.78	3.31	1.96	2.95	late schizogony
MAL7P1.229	7	cytoadherence linked asexual protein	4064	99	56	5.6	0.47	2.79	0.59	1.66	late schizogony
PFA0700c	1	*Plasmodium* exported protein (hyp10)	333	100	18	22.7	6.62	2.76	1.24	1.97	late ring
PF10_0051	10	ADP/ATP carrier protein, putative	995	97	14	5.8	0.32	2.60	0.58	1.49	late ring
PFL2555w	12	*Plasmodium* exported protein (PHISTa)	797	96	11	5.6	0.30	2.49	0.85	1.52	early ring
PFD0980w	4	holo-(acyl-carrier protein) synthase, putative	1785	100	15	3.3	0.44	2.27	1.11	1.78	merozoite
PF11_0014	11	PfMC-2TM_11.1, Maurer's cleft protein	621	89	30	18.3	1.07	2.16	0.79	1.49	late ring
PFA0065w	1	Pfmc-2TM, Maurer's cleft protein	565	100	21	13.6	0.38	2.04	1.70	2.04	late ring
PFC0110w	3	cytoadherence linked asexual protein 3.2	3749	100	144	13.8	0.11	2.02	1.42	1.92	early schizogony
PF07_0124	7	conserved, unknown function	2381	99	14	2.2	0.23	1.97	0.58	1.24	late schizogony
PF07_0042	7	conserved, unknown function	6282	100	103	5.5	0.52	1.97	1.41	1.91	gametocyte
PF11_0344	11	apical membrane antigen 1	1844	100	78	14.1	7.25	1.95	1.52	1.97	late schizogony
PFB0950w	2	conserved *Plasmodium falciparum* protein family	718	100	20	9.7	0.36	1.93	1.31	1.72	merozoite
PF08_0002	8	surface-associated interspersed gene 8.2	5786	94	181	10.6	1.26	1.85	1.55	1.99	gametocyte
PFB0080c	2	*Plasmodium* exported protein (PHISTb)	1133	100	20	6.1	0.92	1.84	0.92	1.40	early ring
PF10_0015	10	acyl-CoA binding protein, isoform 1	273	100	15	19.6	0.35	1.83	1.11	1.60	merozoite
PF07_0004	7	*Plasmodium* exported protein	1472	100	45	10.2	0.56	1.82	0.38	1.06	merozoite
PF07_0085	7	ferrodoxin reductase-like protein	1929	100	10	1.9	0.04	1.82	0.78	1.33	early trophozoite
PF10_0348	10	Duffy binding-like merozoite surface protein	1599	98	32	6.8	1.33	1.81	1.86	2.10	late schizogony
MAL8P1.32	8	nucleoside transporter 2	1758	100	12	2.4	1.39	1.70	−0.14	0.56	early trophozoite
PFA0180w	1	ATP-dependent RNA helicase, putative	3768	100	21	1.2	0.54	1.65	0.22	0.82	early trophozoite
PFE0560c	5	MORN repeat protein, putative	3849	90	20	1.8	0.46	1.63	0.95	1.40	late trophozoite
MAL13P1.105	13	ser/thr protein phosphatise 2A subunit	2512	100	10	1.4	0.03	1.63	0.11	0.68	late trophozoite
PFB0935w	2	cytoadherence-linked asexual protein 2	4264	100	58	4.4	0.16	1.61	1.09	1.49	late schizogony
PFA0665w	1	DBL containing protein, unknown function	6991	80	194	9.5	0.46	1.59	1.35	1.73	null

N, number of aligned nucleotide positions analysed; % CDS, percentage of the complete gene coding sequence analysed; S, number of polymorphic sites analysed per gene; π, pairwise nucleotide diversity index; peak expression, as determined by previous microarray transcriptome analyses; dN/dS, Nei & Gojobori ratio of pairwise nucleotide diversity at nonsynonymous sites compared with synonymous sites. Results for all 2853 genes with 3 or more SNPs are given in Table S2.

Generally, for genes that had been studied previously in endemic African populations by capillary re-sequencing of particular loci (data reported in [14] or from studies reviewed in [13]), there was strong correlation between the Tajima's D values obtained here and those previously reported. Particularly, 11 (92%) out of 12 genes that had positive indices in previous studies also had positive values here: *PF10_0355* (value of 3.31), *ama1* (1.95), *PF10_0348* (1.81), *Pf38/6cys* (1.57), *csp* (1.20), *eba-175* (1.29), *SURFIN4.2* (1.04), *msp7* (1.01), *trap* (0.77), *msp3* (0.09), *sera5* (0.07).

Thirty seven (57%) isolates had mixed genotype infections and 28 (43%) were apparently single-clone infections, as determined by genotyping with highly polymorphic loci *msp1* and *msp2*, similar proportions to those seen in previous studies of clinical isolates at this site [34], [35]. These two separate strata of isolates showed very similar site frequency spectra, with a high correlation of Tajima's D values across all 2853 genes analyzed (Spearman's ρ = 0.62, P<0.0001). The 30 genes having the highest values overall were similarly placed in the top tail of values in both strata, indicating a high level of replication of outlier results (Figure S1).

### Correlations with stages of transcription and particular gene families

**Figure 4:**
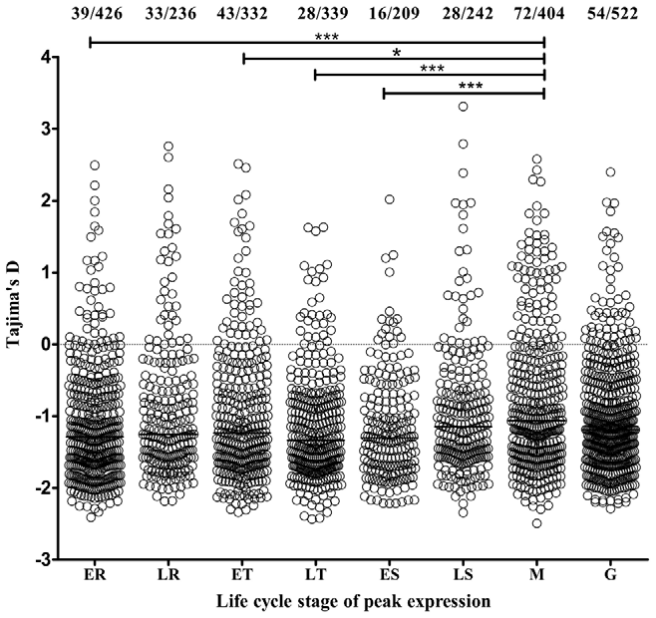
Genes with estimated peak expression at the merozoite stage have highest Tajima's D values overall. Assignment of peak stage transcript expression for 2710 genes in data from microarray studies [37] used an expression time series query implemented by PlasmoDB [36], and are plotted against the polymorphism data from the present study. The points show the values for individual genes (and horizontal bars the medians of all genes) with estimated peak expression at each stage (ER, early ring; LR, late ring; ET, early trophozoite; LT, late trophozoite; ES, early schizont; LS, late schizont; M, merozoite; G, gametocyte). The proportions of genes with values above zero are shown at the top (this is highest for merozoite-stage genes, with 72/404 or 17.8%, p<0.0001 compared with all other genes). Asterisks indicate p values for Mann-Whitney tests on the comparisons of distributions between pairs of stages (* p<0.01, *** p<0.0001).

Transcriptome data from microarray analyses on synchronized parasite asexual blood stages and gametocytes were available (www.plasmodb.org [36], [37]) for 2710 (95.0%) of the 2853 genes with 3 or more SNPs, enabling exploration for associations between stage-specificity of expression and Tajima's D indices of the polymorphic site frequency spectrum (Figure 4). Genes with estimated peak expression in merozoites had higher indices than genes with peak expression at other life cycle stages, significantly for the distribution of values for the merozoite stage compared with four of the other stages separately (Mann-Whitney tests each p<0.01), whereas none of the other stages differed significantly between each other. Tajima's D values were above zero for 17.8% (72 of 404) of merozoite stage peak expression genes, compared with 10.5% (241 of 2306) of those with all other stage peaks (P = 0.0001 after Bonferroni correction for testing each separate stage against the others combined). This indicates that balancing selection is particularly strongly active on this extracellular invasive stage of the parasite in the blood.

**Figure 5:**
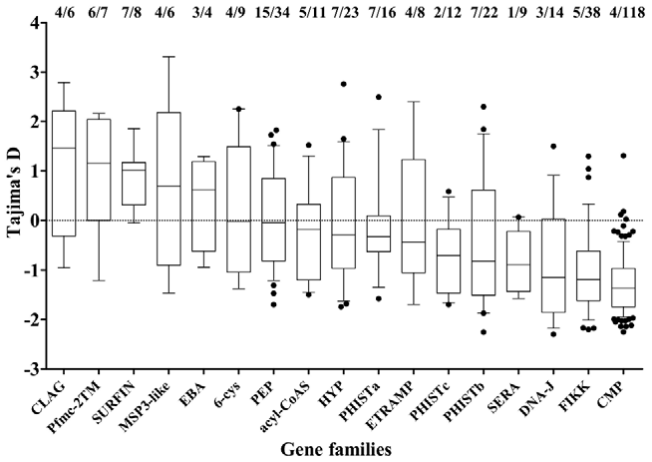
Distribution of Tajima's D values in members of different gene families and groups of genes defined by expression location. For each, plots show the mean (mid-line), one standard deviation (boxes), and 2 standard deviations (whiskers) with any individual outlier genes as points. The proportions of genes with values above zero and the numbers of genes analysed in each gene family are shown above the plot.

Members of small gene families, and others encoding proteins broadly categorized by location of expression were investigated (Figure 5). Most showed a broad range of values of Tajima's D, predominantly negative with a minority positive. The four families with the highest values overall were *clag*, *Pfmc-2TM*, *surfin* and *msp3*-like genes (Figure 5). Highly positive values were also seen for individual genes as diverse as ADP/ATP carrier protein (PF10_0051) and acylCoA synthase (PFD0980w), as well as loci encoding hypothetical proteins of unknown function (Table 1 and Table S2).

### Fine mapping of indices within particular genes

**Figure 6:**
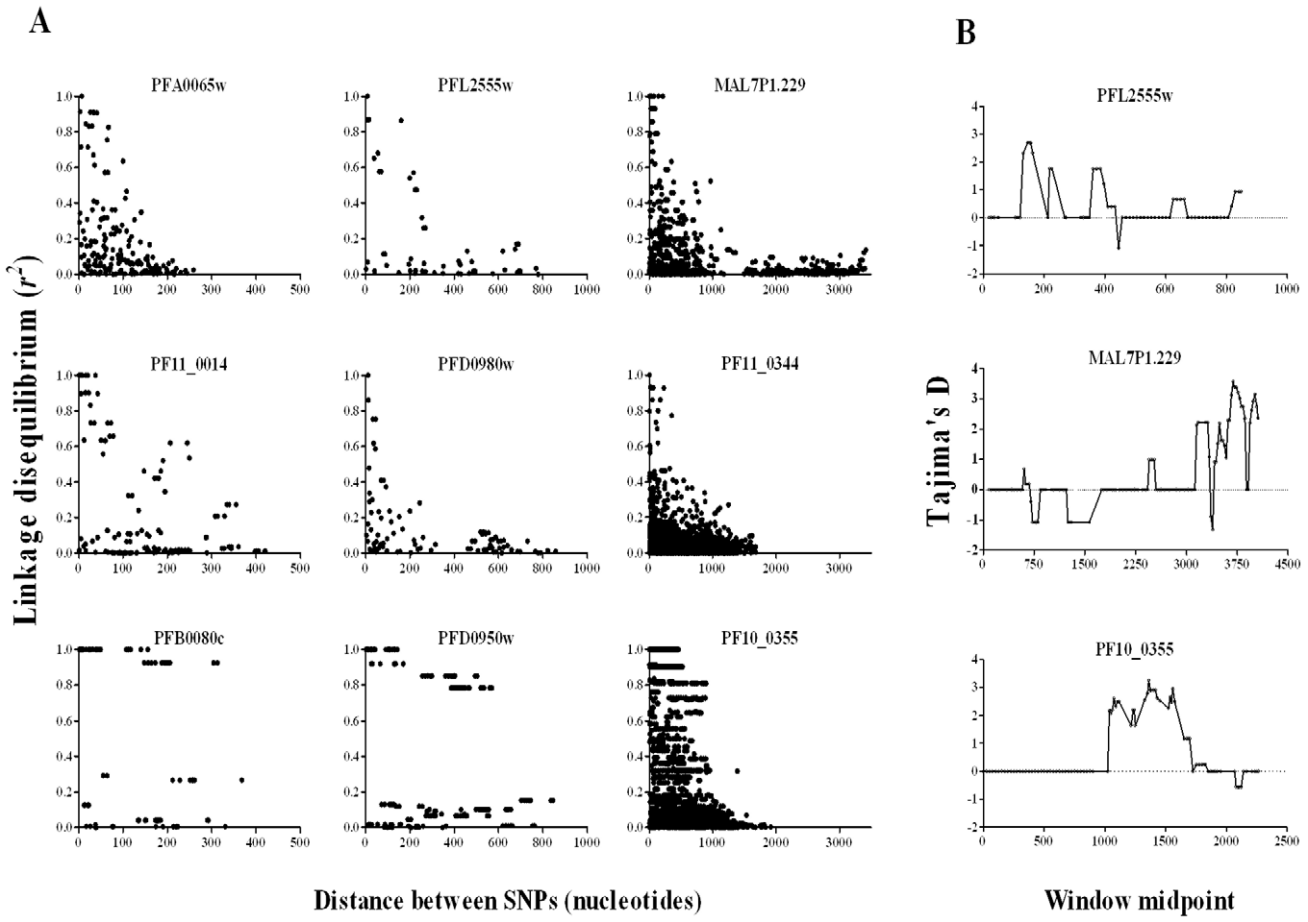
Mapping signatures to particular regions within genes. A. Plots of linkage disequilibrium (*r* ^2^) with distance between polymorphic nucleotides within genes each containing 10 or more SNPs. Nine genes are illustrated: left hand column shows genes with data on SNPs covering <500 bp, middle column 500–1000 bp, and right hand column >1000 bp, each column plotted with a different x-axis scale. Decline of LD with distance is evident in most genes, although the bottom plots show examples with some extended LD over most of the sequence analysed. B. Sliding window analysis identifies regions of genes with candidate signatures of balancing selection: top plot shows a *PHISTa* gene (PFL2555w) with high Tajima's D values in the 5′-region; middle plot shows the strongest signature on a *clag*-like gene (MAL7P1.229) is in the 3′-region; bottom plot for PF10_0355 shows the signature in the middle of the sequence. Window size of 100 bp was applied with step size of 25 bp.

The indices of selection detected were highly locus-specific, as expected where effective recombination rate is very high and linkage disequilibrium (LD) declines rapidly with nucleotide map distance, as expected for most *P. falciparum* populations in Africa [10],[18],[19]. This study was not designed to investigate issues relating to LD in depth, as there is a possibility that some false haplotypes would be derived from consensus sequence contigs generated from mixed genotype infections. Nevertheless, examination of data from genes with 10 or more SNPs was informative even in crude analysis, with very strong LD only generally seen among sites separated by a few hundred nucleotides or less (Figure 6A). A minority of the genes (examples shown in the bottom panels of Figure 6A) showed patterns indicating blocks of sequence that may be in virtually absolute LD, illustrated most clearly for PF10_0355 in which such LD extends for almost 1 kb (as shown previously for this gene with alleles grouping into two major dimorphic forms) [14]. Given that strong LD did not usually persist throughout genes, sliding window analysis was able to reveal heterogeneity of signatures in different parts of a gene (examples shown in Figure 6B). The strongest signature consistent with balancing selection in the *PHISTa* gene PFL2555w is towards the 5′-end (top panel, Figure 6B), whereas for the *clag*-like gene MAL7P1.229 the strongest evidence is near the 3′-end (middle panel, Figure 6B), and for the *msp3*-like PF10_0355 it is in the middle of the gene (bottom panel, 6B).

### Novel pattern of variant expression in a candidate target of balancing selection

**Figure 7:**
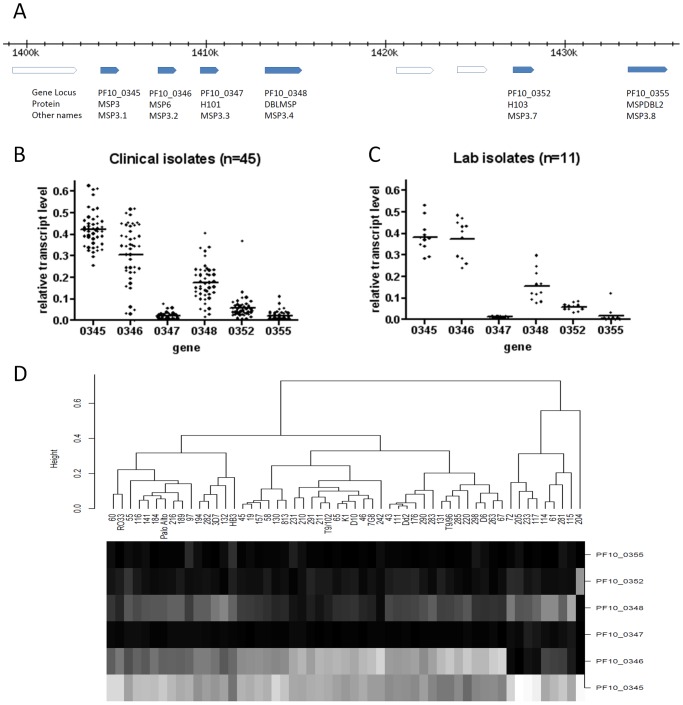
Transcript profiles of the six *msp3*-like genes in *P. falciparum* clinical and laboratory isolates grown to schizont stages. (A) Genomic loci of the six *msp3*-like genes on parasite chromosome 10 (nomenclature and map is based on 3D7 genome sequence version 2.1). Quantitative RT-PCR was based on non-polymorphic sequences (oligonucleotide primers and probes are given in Table S4). (B) Variation in relative transcript levels for the six *msp3*-like genes among 45 Gambian clinical isolates. Relative transcript levels for each gene in each isolate are normalized as a proportion of the sum for all six genes within the isolate. (C) Variation in transcript levels of the genes among 11 diverse laboratory-adapted cultured isolates. (D) Cluster analysis of expression profiles in the 45 clinical isolates and 11 laboratory-adapted isolates. Laboratory isolates are interspersed with the clinical isolates throughout, except for a divergent cluster of only clinical isolates on the right of the figure expressing little or no transcript of the *msp6* gene PF10_0346 (including one isolate that abundantly expressed the *h103*/*msp11* gene PF10_0352).

We investigated PF10_0355 further, as the top hit from the genome-wide analysis. This *msp3*-like gene was originally predicted to encode a protein designated MSPDBL2 (the second merozoite surface protein to have a Duffy-Binding Like domain) [38], also given the designation MSP3.8 [39], and over-expression of the gene by episomal plasmid transfection has conferred reduced sensitivity to culture inhibition by halofantrine [40]. Although most *msp3*-like genes encode proteins associated with merozoites (within schizonts and after extracellular release), microarray and RNA sequence analyses of a few cultured *P. falciparum* lines have previously shown little transcription of PF10_0355 at any developmental stage [37], [41], [42], [43]. To survey transcript profiles of the six *msp3*-like genes (Figure 7A), 45 Gambian clinical *P. falciparum* isolates cultured *ex vivo* to schizont stage were assayed by quantitative RT-PCR (Figure 7B). The *msp3* gene (PF10_0345) was expressed in all isolates, while *msp6* (PF10_0346) and *dblmsp* (PF10_0348) were expressed in most isolates at varying levels, and the other three genes (including PF10_0355) showed relatively low transcript levels in almost all isolates. Schizont stage cultures of 11 long term culture-adapted parasite lines of diverse origin were assayed (Figure 7C), showing a similar range of expression for each gene as observed among clinical isolates. It is notable that the *h103* gene (PF10_0352), which has also been named as *msp11*, was highly transcribed in one clinical isolate only. Cluster analysis of the transcript profiles showed the laboratory and clinical isolates interspersed with each other (Figure 7D), and levels of PF10_0355 transcript were very low in all except clinical isolate 97 and laboratory clone HB3.

**Figure 8:**
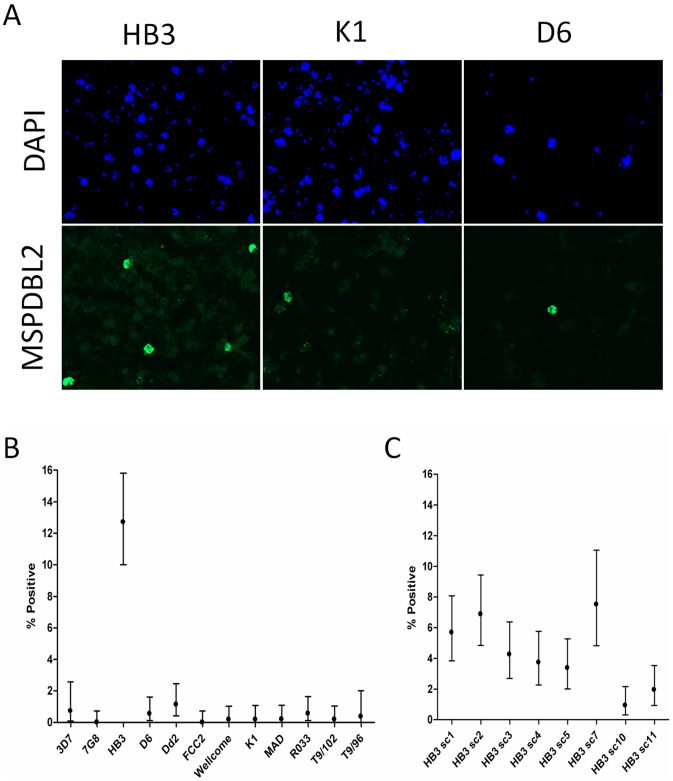
Immunofluorescent FITC labelling of the MSPDBL2 antigen. (A) Immunofluorescent FITC (green) labelling of the MSPDBL2 antigen (product of PF10_0355) in a minority of schizonts with individual microscopic fields illustrated for three parasite isolates (HB3, K1 and D6), alongside staining of parasite DNA by DAPI (blue) for the same fields. Parasite immunofluorescence shows reactivity with 1/500 diluted mouse antiserum raised to a recombinant protein representing the N-terminal of MSPDBL2. (B) Proportions (with 95% CI) of schizonts positive for DBLMSP2 in 12 cultured isolates each with a different single parasite genotype. (C) Proportions of schizonts positive for DBLMSP2 in eight sub-clones of clone HB3. Exact numbers counted are shown in Table S3.

To investigate protein expression in schizonts, 12 genetically distinct parasite lines that were each apparently clonal were studied by immunofluorescence microscopy with antibodies raised against recombinant proteins based on conserved parts of the product of PF10_0355 (Figure S2). Remarkably, antibodies to the PF10_0355 product reacted against only a small minority of mature schizonts (Figure 8A). Immature parasite stages including early schizonts with <8 nuclei were all negative, so each parasite line was scored by counting several hundred mature schizonts (with at least 8 DAPI-stained nuclei), showing that ∼1% or less were positive in each line, with the exception of HB3 in which 12.7% (68/535; 95% CI, 10.0–15.8%) were positive (Figure 8B, and Table S3). To test for stability of proportions positive, HB3 was grown again from cryopreserved stock, and mature schizonts tested after approximately 2 weeks of independent culturing. These showed a MSPDBL2-positive proportion of 9.1% (52/574; 95% CI, 6.8%–11.1%), marginally lower than seen in the previous culture (P = 0.051). To test for changes over a longer period of independent culturing, a panel of 8 sub-clones of HB3 that had been cultured separately for an average of 4 months (∼60 replicative cycles) was then assayed. Among these sub-clones, the proportions of mature schizonts positive showed a spectrum ranging from 0.9% (5/533; 95% CI, 0.3–2.2%) to 7.5% (23/306; 95% CI, 4.8–11.1%) (Figure 8C).

Multiple-labelling immunofluorescence assays were then performed on the panel of 12 different parasite lines using antibodies against conserved sequences of MSP3 (product of gene PF10_0345), MSP6 (PF10_0346), and DBLMSP (PF10_0348). This indicated no mutual exclusion between MSPDBL2 and the other more commonly-expressed MSP3-like proteins, with MSPDBL2-positive schizonts being positive for MSP3, MSP6 and DBLMSP in each of the lines (Figure S3). A particular histone methylation mark H3K9me3 (tri-methylation of lysine at residue 9 of H3) is a feature of sub-telomeric antigenic variant genes in their repressed state, and PF10_0355 is one of only very few genes elsewhere in the genome to have such a heterochromatic signature, apparent also on flanking sequences but not extending to the other *msp3*-like paralogues [44]. In contrast to the anti-MSPDBL2 antibodies, anti-MSP3, anti-MSP6 and anti-DBLMSP antibodies reacted against most mature schizonts in every isolate studied here, with an exception that DBLMSP expression was absent in the RO33 line (as expected from the existence of a stop codon in the PF10_0348 gene in this line only) [17].

## Discussion

This population genomic analysis of *P. falciparum* in a single endemic location has indentified many new genes with polymorphic site frequency spectra consistent with balancing selection, as well as confirming results for previously studied candidate antigen genes. These genes appeared as exceptions against a genomic background in which most genes had negative values of Tajima's D, as expected from historical population expansion [45] and also seen with the mitochondrial genome [46]. As all parasite isolates sequenced were collected in one transmission season from a single area in the Gambia we minimized population structuring in the sample, and were able to identify genes in the outlying skewed tail of strongly positive values of Tajima's D and other supporting indices. Not all individual genes with high values of Tajima's D will be under balancing selection, as there is likely to be a wide range of values under neutrality due to genetic drift variance among loci, as well as sampling variance affecting the values for genes with few SNPs. The analysis should therefore be regarded as a screen to identify potential candidates under balancing selection, in which all hits require validation. Earlier data on polymorphism among small numbers of *P. falciparum* lines and a partial sequence of the chimpanzee parasite *P. reichenowi* allowed preliminary survey of diversity-versus-divergence indices including McDonald-Kreitman and Hudson-Kreitman-Aguade ratios [20], [22], and comprehensive analyses to derive such indices from our population-based data will be useful after a more complete draft of the *P. reichenowi* genome sequence is published.

Analyses here considered the consensus sequence for each gene in each isolate, as this could be clearly determined using available methods. Results were similar for subsets of isolates that respectively contained apparent single or multiple parasite clones, on the basis of a routine genotypic screen. It is not currently possible to resolve parasite genomic haplotypes within mixed genotype infections [23], unless they are cloned and cultured long term *in vitro* [47] during which artificial selection may occur. Although haplotype resolution was not necessary for the current analyses, development of future methods to achieve this could allow investigation of processes of within-isolate selection among parasites, which have a spectrum of relatedness due to mixed inbreeding and outbreeding [47], [48].

As expected from the high recombination rate in this parasite, patterns of polymorphism consistent with balancing selection were tightly localized within individual genes, whereas in organisms with lower recombination rate balancing selection often affects polymorphism at flanking loci [49]. Tajima's D values were highest for genes with estimated peak expression in merozoites, indicating that exceptionally strong balancing selection operates on this extracellular stage, consistent with likely effects of acquired immunity or interaction with diverse erythrocyte receptors for invasion. There were high values for previously studied antigen genes, such as the apical membrane antigen 1 gene (*ama1*) which encodes a prime vaccine candidate [27] previously shown to be under balancing selection in several independent studies of different populations [17], [25], [29], [30], [31].

The gene with the highest Tajima's D value overall was the *msp3*-like *PF10_0355* that also had the highest value among the candidate genes studied previously [14]. Recent data have indicated differences in transcript levels of this gene among some parasite lines [40], and existence of a protein product on the merozoite surface [39]. Our results significantly extend these findings to show that this merozoite protein is expressed in only a minority of mature schizonts within any parasite clone, but the proportion of positive schizonts varies significantly among clones, and also varies over time for a single clone and among sub-clones cultured separately. There has been considerable characterization of antigenic variation caused by large sub-telomeric gene families expressing proteins on the infected erythrocyte surface [50]. The merozoite protein genes for which variant expression has previously been described are also sub-telomeric [51], [52], [53], [54], whereas the PF10_0355 gene is exceptional in showing the H3K9me3 heterochromatic marking associated with silencing of sub-telomeric genes, but in a more centromeric position [44]. Over-expression of the gene on an episomal plasmid (free from heterochromatin-associated repression) conferred resilience of *in vitro* growth in the presence of halofantrine through an unknown mechanism [40]. If this protein directly affects parasite growth in differing environments, this could potentially contribute to a system of balanced polymorphism and repression of expression. It would also suggest that identifying its importance as a target of naturally-acquired immunity might be more demanding than has been the case for other merozoite antigens [55].

A *clag*-like gene (*MAL7P1.229*) had the second highest Tajima's D value in the genome, and the family of *clag* genes that encode merozoite rhoptry proteins [56] also generally ranked highest, although values for *clag 3.1* and the adjacent gene *clag 3.2* may be affected to some extent by gene conversion [57]. Particular *clag* genes have alternative expression patterns between parasites within a single clone [51], suggesting that structural polymorphism under balancing selection may also be associated with variant expression. Members of the *Pf-mc-2TM* family encoding proteins associated with Maurer's clefts also had very high values (most exceptionally for *PF11_0014* and *PFA0065w*), and members of this family have been shown to have clonally variant expression [58]. The families of *surfin* and *eba* genes also ranked highly, and each contain members that have previously shown patterns of polymorphism suggesting balancing selection [14], [59], [60] and exhibit variable expression among parasite isolates [35], [40], [61], [62].

Polymorphic site frequency spectra consistent with balancing selection were also seen in some exported protein genes, including members of the *HYP* and *PHIST* families that have transmembrane domains or signal peptides [63]. Individual members of all three classes of the *PHIST* gene family had high values of Tajima's D, of which the highest were for particular *PHISTa* (PFL2555w) and *PHISTb* genes (PFD1170c and PFB0080c). Allelic polymorphisms in *HYP* and *PHIST* genes are likely to contribute to observed phenotypic diversity in clinical isolates, alongside effects of variant expression [62], [64], [65].

These results suggest that many targets of balancing selection may also undergo phase variation. We consider that immune selection is likely to be the primary cause of such selection on asexual haploid blood stage parasites [60], [66], [67], [68], but other mechanisms may operate on some genes, including interactions with genetically polymorphic host cell receptors that are themselves under balancing selection [69], [70], or hypothetical systems of non-self recognition among genetically heterologous asexual parasites within infections [71]. At other stages of the life-cycle, it is possible that balancing selection is driven by non-self recognition among parasite gametes that are transmitted to mosquitoes, or heterozygote advantage operating at the very brief diploid stage in the mosquito midgut. Further work is needed to determine causes of selection on most of the affected genes highlighted, and it is preferable to first perform population genetic analyses in other endemic populations to test initial inference of selection for individual genes emerging from this study. Similar approaches should also be effective in identifying candidate targets of balancing selection in the genomes of other eukaryotic pathogens, including other malaria parasite species.

## Materials and Methods

### Ethics statement

Ethical approval for the study was obtained from the Gambia Government and MRC Joint Ethics Committee, and the Ethics Committee of the London School of Hygiene and Tropical Medicine. Written informed consent was obtained from a parent or guardian of each child contributing a blood sample. In addition, assent was obtained from children over 10 years of age. Following review (LSHTM Approval PF-486), antibodies were obtained commercially under commercial sub-contract, and all animal work protocols were approved and licensed by the UK Home Office as governed by law under the Animals (Scientific Procedures) Act 1986 (Project licenses 70/7051 and 80/2061). The animals were handled in strict accordance with the “Code of Practice Part 1 for the housing and care of animals (21/03/05)” available at http://www.homeoffice.gov.uk/science-research/animal-research/, and the numbers used were the minimum consistent with obtaining scientifically valid data.

### Malaria patients and *P. falciparum* isolates

Patients with *P. falciparum* malaria were recruited in the malaria season between August and December 2008 from four health facilities located within a radius of 20 km in the coastal area of The Gambia (Royal Victoria Teaching Hospital in Banjul, the MRC clinic in Fajara, Jammeh Foundation for Peace Hospital in Serekunda, and Brikama Health Centre). All recruited malaria cases had a temperature of >37.5°C on presentation or history of fever in the previous 48 hours, and a minimum of 5000 *P. falciparum* parasites µl^−1^ estimated by thick film examination. A thin blood smear confirmed each infection as *P. falciparum* only. After informed consent, and under approval by the Joint Gambian Government and MRC Ethics Committee, up to 5 ml of venous blood was collected from each subject in heparinised tubes immediately prior to treatment. Plasma was removed from blood samples after centrifugation, and erythrocytes were separated from leukocytes by Nycoprep density gradient centrifugation, washed and re-suspended at 50% haematocrit in incomplete RPMI medium. Samples were further processed to deplete human leukocytes, either by filtration of cell suspension through Plasmodipur filters, or by sedimentation on plasmagel followed by magnetic capture using anti-HLA antibody-coated beads. Following separation, leukocyte-depleted erythrocytes were washed in incomplete RPMI 1640 and stored at −80°C. DNA was assayed for presence of single or multiple clones of *P. falciparum* by genotyping the highly polymorphic repeat loci in *msp1* and *msp2* [72]. For analysis of gene expression, forty five clinical isolates of *P. falciparum* cultured *ex vivo* to the first generation schizont stage were analyzed from samples collected over three previous malaria seasons (2005–2007) in The Gambia [35], [73]. Fourteen laboratory-adapted *P. falciparum* isolates of diverse origin were cultured separately in London: 3D7, cloned from an airport malaria case in The Netherlands; D6, RO33 and Palo Alto, from Africa; FCR3 and Wellcome, nominally from Africa but each suspected to have been contaminated and clonally replaced by different parasites more than 20 years ago during culture; K1, T9/96, T9/102, Dd2, FCC2 and D10, from Southeast Asia; HB3 from Honduras; 7G8 from Brazil.

### DNA processing and sequencing

Parasite DNA was extracted from 400 µl of packed erythrocytes from each sample using QIAamp DNA blood midi kit (Qiagen, United Kingdom). The ratio of human to parasite DNA was then determined by quantitative PCR assays on parasite apical membrane antigen 1 gene (*ama1*, following published protocol [74]) and human RnaseP gene (Applied Biosystems protocol). Sixty eight processed samples containing over 50% parasite DNA (<50% human DNA) were selected as potentially suitable for Illumina paired-end shotgun sequencing, and standard Illumina sequencing libraries were prepared following the manufacturer's recommended protocol. Short paired-end reads (37 or 76 base pairs) were generated and mapped onto the *P. falciparum* 3D7 reference genome sequence (version 2.1, June 2010) using the Burrows-Wheeler Aligner (BWA) program, with an algorithm that allowed for polymorphic positions (>98.5% matched excluding indels [75]). The sequence read data have been made available at the European Nucleotide Archive http://www.ebi.ac.uk/ena/data/view/ERP000190, and individual sample ID numbers are given in Table S1. To generate consensus contiguous sequences for each gene per isolate, majority reads were assembled across each coding sequence [76], using SAMtools to generate a read pileup. Sixty-five of the isolates (Table S1) had coverage of above 80% of all coding sequences with mean read depth of at least 10; three other isolates had lower read coverage and were not analysed further. The consensus majority read sequence for each gene in each isolate was analyzed as a sampled allele sequence, which would correspond in almost all cases to the actual allele of the single or most abundant clone in the infection. We excluded genes from three hypervariable families (*var*, *rifin* and *stevor*) and any other individual genes that did not have more than 70% coverage for at least 50 isolates at a read depth of 5 or more. An R script automating analysis with Tandem Repeat Masker and Muscle 3.6 software was used to mask repeats and re-align non-repetitive sequences for each of the genes analysed. Poorly aligned contig sequences were checked and removed using BioEdit (http://www.mbio.ncsu.edu/bioedit/bioedit.html).

### RNA extraction and quantitative transcript analysis

Cultures of parasites predominantly at schizont stage were mixed with four volumes of TRIzol Reagent (Ambion), and aliquots stored at −80°C for subsequent RNA extraction using RNeasy Micro (Qiagen, UK). RNA concentration and purity were determined using a NanoDrop ND-1000, and mRNA was reverse-transcribed with Oligo-dT using TaqMan reverse transcription reagents (Applied Biosystems, UK). For real-time PCR-based transcript quantification, cDNA was assayed in a fluorogenic 5′ nuclease assay (TaqMan chemistry) on a Rotor-Gene 3000 (Corbett Life Sciences), with gene-specific TaqMan primers and probe sets based on non-polymorphic unique sequences within each of the six *msp3*-like genes (Applied Biosystems) (Table S4), and primers and probes for *ama1* based on those previously described [74]. All probes were labeled with 6-carboxy-fluorescein (FAM) on the 5′-end and a non-fluorescent quencher (MGB-NFQ, Applied Biosystems) on the 3′-end and used in single reporter assays. Reactions were carried out in 25 µl volumes using 900 nM of each primer and 250 nM of probe, with one cycle at 50°C for 2 min and 95°C for 10 min, followed by 40 cycles of 95°C for 15 s and 60°C for 1 min. Each run included controls and a standard curve based on 10-fold dilutions of 3D7 genomic DNA.

### Cloning and expression of MSPDBL2 recombinant antigens

Two constructs were designed to express parts of the N- and C-terminal regions of the MSPDBL2 (MSP3.8) product of PF10_0355, as glutathione S-transferase (GST)-tagged proteins in *E. coli* (Figure 3A). Sequences corresponding to nucleotide positions 70–273 and 1615–1770 of the PF10_0355 gene, flanking the central region encoding the DBL domain (Figure S2), were PCR amplified from 3D7 genomic DNA, cloned into the pGEM Easy TA vector (Promega), and sequence verified. Correct sequence inserts were subcloned into the pGEX-2T expression vector (GE Healthcare), sequenced again to ensure fidelity and transformed into BL21(DE3) *E. coli* cells for expression. Expression and affinity purification was performed as described previously for other GST-fusion proteins [77]. Products were visualised by SDS-PAGE and assayed for antigenic reactivity to IgG in sera from a panel of Gambian adults (Figure S2).

### Immunofluorescence assays (IFA)

Antibody reactivities of murine antisera raised to the MSPDBL2 recombinant proteins, a rabbit antiserum to a conserved part of MSP3 (codons 234–354 of PF10_0345) [60], and a rat antiserum to a conserved part of MSP6 (codons 198–255 of PF10_0346), were tested against different *P. falciparum* lines using immunofluorescence assays (IFA). Parasite cultures with a large proportion of schizonts were washed in PBS/1% BSA, resuspended to 2.5% hematocrit and 15 µl aliquots spotted onto multiwell slides (Hendley, Essex, UK) which were then air-dried and stored at −40°C with desiccant until required. Following a recommended fixation protocol [78], slides were bathed in 4% paraformaldehyde in PBS for 30 min, followed by 10 min in 0.1% Triton X-100 in PBS and then overnight at 4°C in PBS/3% BSA. After air-drying, wells were incubated with defined dilutions of each test serum (including initial serial doubling dilutions from 1/200 to 1/409600) in PBS/3% BSA and incubated for 30 min at room temperature. Slides were rinsed 3 times in PBS, excess wash buffer removed and wells incubated for 30 min with a 1/500 dilution of biotinylated anti-mouse IgG (Vector Laboratories, USA) in PBS/3% BSA, washed 3 times in PBS, and incubated for 30 min with 1/500 dilution of fluorescein strepavidin (Vector Laboratories). Mounting fluid with DAPI (Vectashield, Vector Laboratories) was added and each slide sealed with a cover slip. For triple-labelled IFA, selected individual murine antibodies raised to the PF10_0348 and PF10_0355 N-terminal conserved antigens were used, followed by incubation with rhodamine-labelled anti-mouse Ig (at 1/250 dilution). To the same wells rabbit anti-MSP3 conserved antigen raised to the C-terminal region of MSP3 was added followed by 1/250 dilution of FP642-labelled anti-rabbit Ig (FluoProbes, Interchim, France). Finally, rat anti-MSP6 was added followed by 1/500 dilution of FITC labelled anti-rat Ig (Jackson laboratories, USA). All antigen-specific sera were used at 1/400. For doubled-labelled IFA, the protocol was as outlined except that rabbit antiserum raised to the PF10_0348 N-terminal conserved region was used alongside mouse antiserum to the PF10_0355 N-terminal conserved region.

### Antibodies against recombinant proteins

Antibodies to recombinant proteins were obtained commercially by immunization of a small number of laboratory animals as reagents to characterize native parasite proteins, with all protocols and practices approved and licensed by the UK Home Office as governed under the Animals (Scientific Procedures) Act 1986. Numbers of animals immunized were the minimum to reasonably ensure that at least one animal would produce adequate titer antibodies to each protein. Five CD1 outbred mice were immunized with 25 µg of each of the N- and C-terminal MSPDBL2 recombinant antigens emulsified in Freund's complete adjuvant delivered subcutaneously, and boosting immunizations were performed twice at 28 day intervals in Freund's incomplete adjuvant. Sera were collected before immunization and final serum collection made 7 days after the last immunization (Pharmidex, UK). Purified recombinant MSP3 conserved antigen was used to immunize three New Zealand white rabbits with each receiving 200 µg doses of purified protein emulsified in Freund's adjuvant. Following a primary intramuscular immunization in Freund's complete adjuvant, booster immunizations were given in Freund's incomplete on days 14, 28, 42, 56 and 70. Sera were collected before immunization and final serum collection made 7 days after the last immunization (Pettingill Technology Ltd, UK). Two Sprague-Dawley rats were immunized with 25 µg doses of purified recombinant MSP6 protein, with Freund's complete adjuvant for primary immunization and Freund's incomplete adjuvant for three boosting doses at intervals of 7 days, with final serum collection made 7 days after the last immunization (Harlan, UK).

### Statistical analyses of DNA polymorphism

Summary statistics and neutrality tests based on the polymorphic nucleotide site frequency spectra were calculated using Variscan 2.0 [79]. Tajima's D test takes into account the average pairwise nucleotide diversity between sequences (*π*) and the population nucleotide diversity parameter (Watterson's *θ*_w_) expected under neutrality from the total number of segregating sites for a population at mutation-drift equilibrium [80], with positive values (when *π*>*θ*_w_) indicating an excess of intermediate frequency polymorphisms and negative values indicating an excess of rare polymorphisms. Fu and Li's test statistics D* and F* are based on the difference between the observed number of singleton nucleotide polymorphisms and the number expected under neutrality given estimates of nucleotide diversity from *θ*_w_ and *π*, for D* and F* respectively [81]. An R script was employed to automatically run Variscan 2.0 on all masked gene coding sequence alignments generated above, on a module that included only sites from alignments in which at least 50 out of the 65 isolates had nucleotide calls. Output was transformed into tables with a Matlab script and filtered to include only genes for which >70% of non-repeat nucleotide sites were confidently aligned. Gene alignment data meeting these criteria were further analysed for summary indices of allele frequency distributions and linkage disequilibrium using DnaSP 5.1 [82] to check for concordance of results obtained with Variscan 2.0 and perform additional tests including calculation of dN/dS ratios.

Mann-Whitney tests were used to assess significance of differences in distribution of values of indices across different gene categories. Peak stage of gene transcript expression in published microarray data was assessed using an expression time series query in PlasmoDB (http://plasmodb.org/plasmo/) [36], [37]. Correlations between pairs of indices were analysed by Pearson's correlation coefficient, and comparisons between proportions of categorical variables were performed by Chisquare tests.

### Statistical analyses on gene transcript data

Transcript levels derived by quantitative reverse transcriptase PCR, as described above for each of the 6 *msp3*-like genes, were normalized as a proportion of the sum of the transcript levels for these genes within each isolate. Expression profiles were generated using a heat map representing the relative proportions of each transcript using the Bioconductor suite in R, and Ward's clustering was applied to derive a hierarchical cluster analysis of isolate expression profiles, in which each object is initially assigned to its own cluster and then the algorithm proceeds iteratively by continually joining the two most similar clusters (dissimilarities between clusters are the squared Euclidian distances between cluster means). Spearman's rank nonparametric correlation coefficient was used to measure the correlation between relative levels of expression. Mann-Whitney *U* tests were used to assess whether there were significant differences between dichotomous groups in the distributions of continuous variables including the relative amounts of each of the transcripts. Statistical analysis was performed using Stata version 9.0 or 11.0 software, and plots were generated using GraphPad Prism version 4.02 software.

## Supporting Information

Figure S1 Spectrum of values of Tajima's D and Fu & Li's F* indices for genes with 3 or more SNPs analysed separately for multiple clone isolates (n = 37) and single clone isolates (n = 28). The correlation for Tajima's D values across all genes between the two strata is highly significant (Spearman's ρ = 0.62, P<0.0001). The genes with the top 30 values of Tajima's D in the overall analysis of 65 isolates are shaded in red, and are at the top tail of the distribution within each of the independent strata of samples.
(PDF)

Figure S2 Recombinant proteins based on conserved sequences in the N-terminal and C-terminal regions of the PF10_0355 product MSPDBL2. A. The position of the sequences are shown as bars underneath the scheme of MSLDBL2 (black shading indicates the DBL-domain, grey shading the SPAM domain, and hatched shading the main repeat sequence). B. SDS-PAGE gel showing the *E. coli*-expressed GST-fusion proteins. C. ELISA data showing antibody reactivity in a panel of 39 Gambian adults, with strong correlation between the reactivity to N- and C-terminal regions (Pearson's r = 0.94). Dashed lines show the cut-off OD values to determine positivity (mean +3SD of OD values of a panel of 20 sera from individuals in the UK who had not been exposed to malaria). Fourteen (36%) of the Gambian adults had positive antibody reactivity to both proteins.
(PDF)

Figure S3 Multiple-labelled immunoflourescence showing that the minority of parasites expressing MSPDBL2 (product of PF10_0355) also express other MSP3-like proteins. Three parasite lines are illustrated out of 12 tested, with parasites stained with DAPI (blue) for DNA, rhodamine (red) for antibodies to MSPDBL2 (N-terminal), FP642 (purple) for antibodies to MSP3, FITC (green) for antibodies to MSP6. In separate assays, parasites that reacted with antibodies to MSPDBL2 also reacted with antibodies to DBLMSP (product of PF10_0348), but many parasites positive for DBLMSP were negative for MSPDBL2 as expected (not shown).
(PDF)

Table S1 List of 65 Gambian *P. falciparum* isolates analysed with details of individual sample material, read coverage and accession numbers for archived short-read sequences.
(XLSX)

Table S2 Summary indices of polymorphic site frequency spectra in each of 2853 genes with ≥3 SNPs in a Gambian population sample of *P. falciparum* clinical isolates (n = 65)
(XLSX)

Table S3 Exact counts of mature schizonts positive for MSPDBL2 (antibody to N-terminal) by immunofluorescence in each parasite line. Similarly low proportions of parasites were seen reactive with antibodies to the C-terminal although fewer parasites were counted (data not shown).
(PDF)

Table S4 Sequences of primers and probes, and assay conditions used for quantitative real time PCR to assay transcript abundance of each of the six *msp3*-like genes.
(PDF)
