# Supplementary figures and images for: Population Genomic Scan for Candidate Signatures of Balancing Selection to Guide Antigen Characterization in Malaria Parasites
Source: PLoS Genet. 2012 Nov 1;8(11):e1002992. doi: 10.1371/journal.pgen.1002992 (PMC3486833; doi:10.1371/journal.pgen.1002992)

## Multiple clone isolates (n = 37)

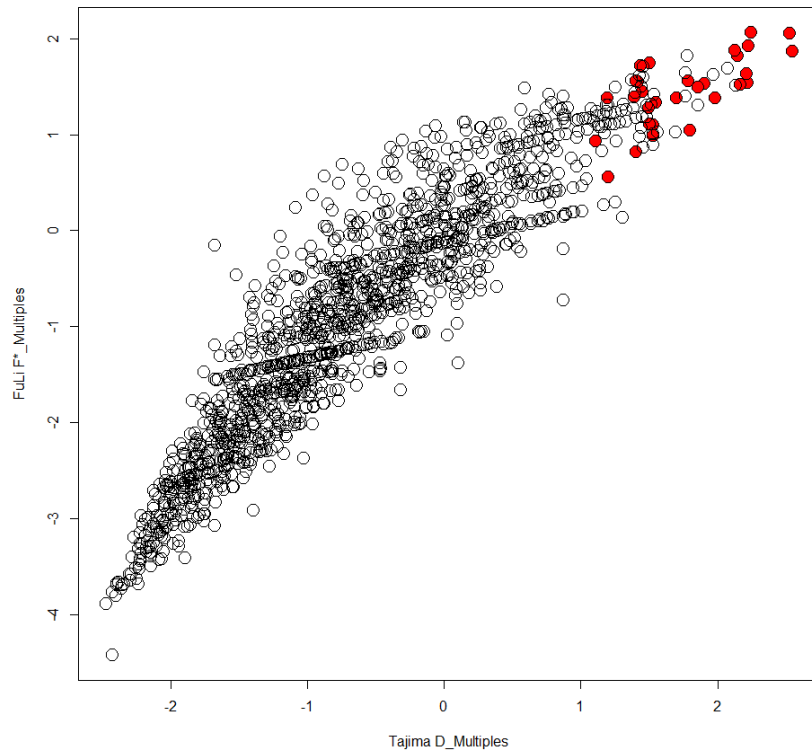

## Single clone isolates (n = 28)

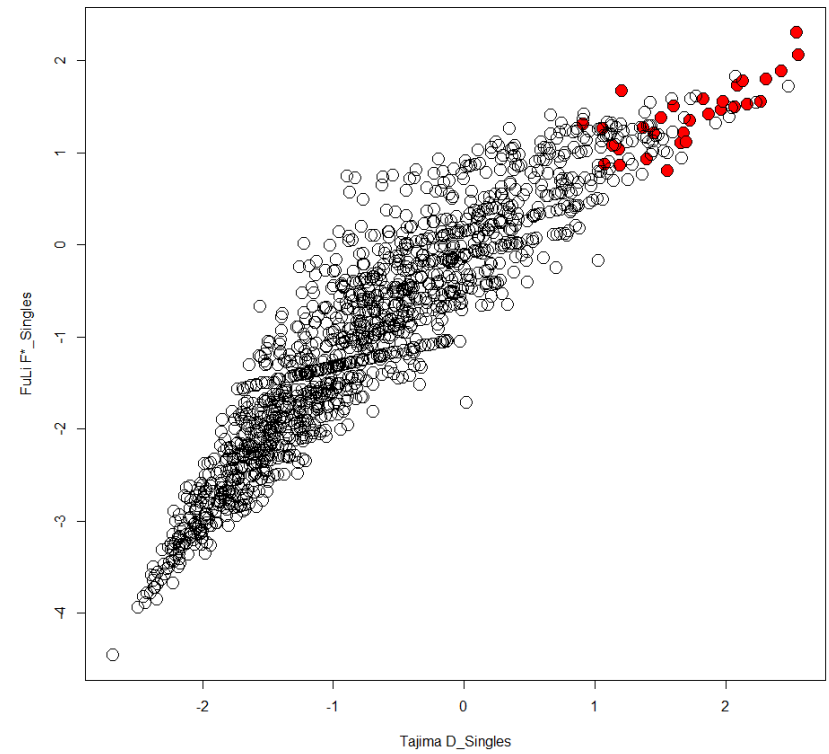

Supplement: Figure S1 — Spectrum of values of Tajima's D and Fu & Li's F* indices for genes with 3 or more SNPs analysed separately for multiple clone isolates (n = 37) and single clone isolates (n = 28). The correlation for Tajima's D values across all genes between the two strata is highly significant (Spearman's ρ = 0.62, P<0.0001). The genes with the top 30 values of Tajima's D in the overall analysis of 65 isolates are shaded in red, and are at the top tail of the distribution within each of the independent strata of samples. (PDF) [file pgen.1002992.s001.pdf]

A

PF10\_0355  
MSPDBL2

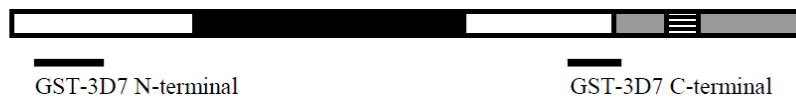

B

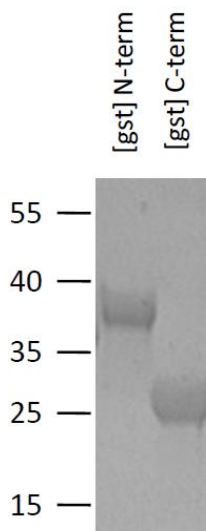

C

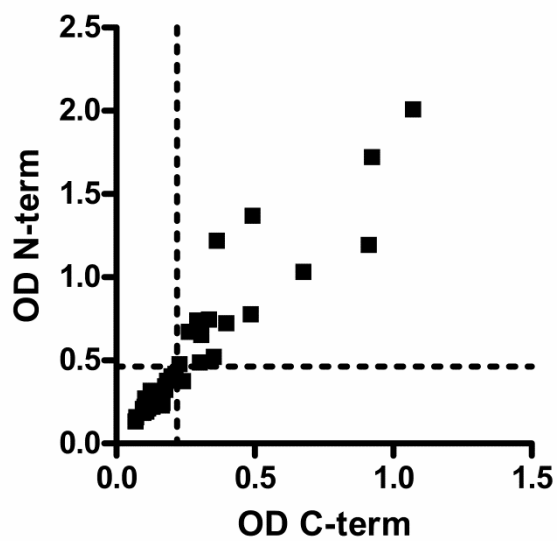

Supplement: Figure S2 — Recombinant proteins based on conserved sequences in the N-terminal and C-terminal regions of the PF10_0355 product MSPDBL2. A. The position of the sequences are shown as bars underneath the scheme of MSLDBL2 (black shading indicates the DBL-domain, grey shading the SPAM domain, and hatched shading the main repeat sequence). B. SDS-PAGE gel showing the E. coli-expressed GST-fusion proteins. C. ELISA data showing antibody reactivity in a panel of 39 Gambian adults, with strong correlation between the reactivity to N- and C-terminal regions (Pearson's r = 0.94). Dashed lines show the cut-off OD values to determine positivity (mean +3SD of OD values of a panel of 20 sera from individuals in the UK who had not been exposed to malaria). Fourteen (36%) of the Gambian adults had positive antibody reactivity to both proteins. (PDF) [file pgen.1002992.s002.pdf]

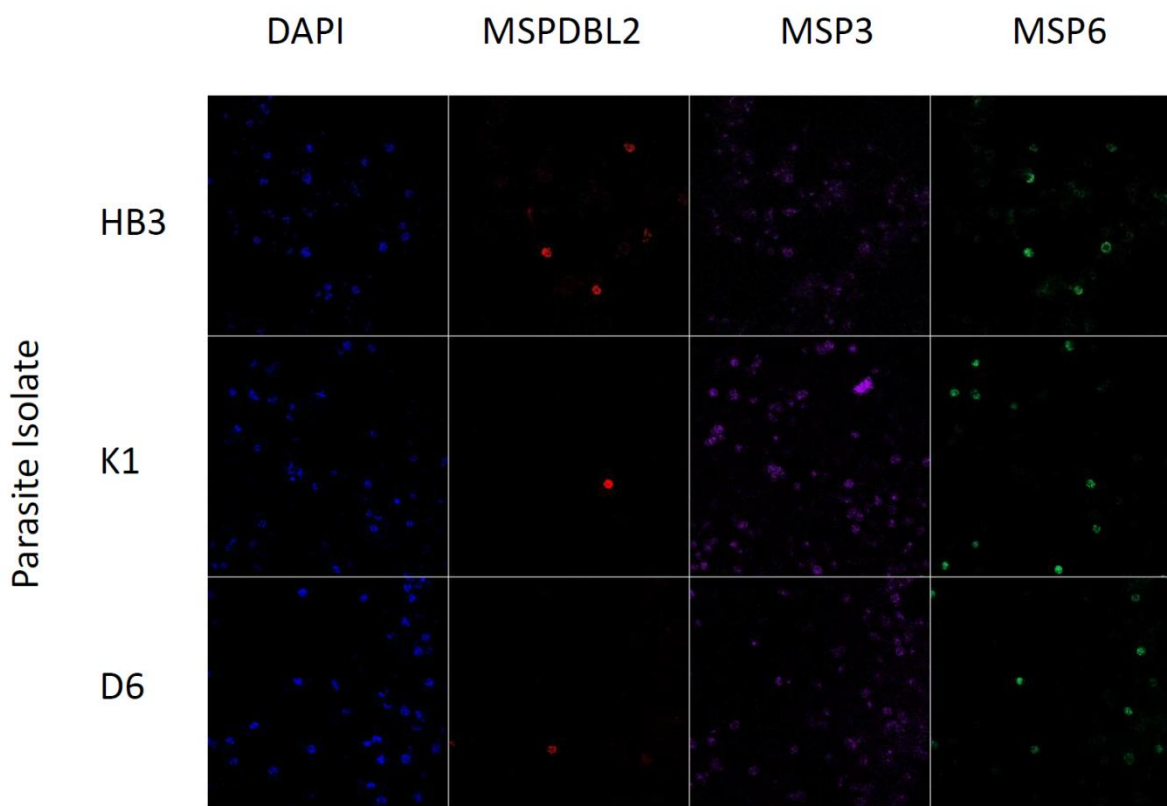

Supplement: Figure S3 — Multiple-labelled immunoflourescence showing that the minority of parasites expressing MSPDBL2 (product of PF10_0355) also express other MSP3-like proteins. Three parasite lines are illustrated out of 12 tested, with parasites stained with DAPI (blue) for DNA, rhodamine (red) for antibodies to MSPDBL2 (N-terminal), FP642 (purple) for antibodies to MSP3, FITC (green) for antibodies to MSP6. In separate assays, parasites that reacted with antibodies to MSPDBL2 also reacted with antibodies to DBLMSP (product of PF10_0348), but many parasites positive for DBLMSP were negative for MSPDBL2 as expected (not shown). (PDF) [file pgen.1002992.s003.pdf]
