# Supplementary material for: Population Genomic Scan for Candidate Signatures of Balancing Selection to Guide Antigen Characterization in Malaria Parasites
Source: PLoS Genet. 2012 Nov 1;8(11):e1002992. doi: 10.1371/journal.pgen.1002992 (PMC3486833; doi:10.1371/journal.pgen.1002992)
Supplement: Table S3 — Exact counts of mature schizonts positive for MSPDBL2 (antibody to N-terminal) by immunofluorescence in each parasite line. Similarly low proportions of parasites were seen reactive with antibodies to the C-terminal although fewer parasites were counted (data not shown). (PDF) [file pgen.1002992.s006.pdf]

Supplementary Table S3. Exact counts of mature schizonts positive for MSPDBL2 (murine antibody to N-terminal region) by immunofluorescence in each parasite line.

Parasite isolate/clone	Number of mature schizonts counted	Number positive for MSPDBL2	Percentage positive for MSPDBL2
Isolate/clone:			
HB3	535	68	12.71
3D7	278	2	0.72
T9/96	275	1	0.36
Dd2	530	6	1.13
Wellcome	541	1	0.19
FCC2	>5000	0	0
7G8	>5000	0	0
D6	538	3	0.56
K1	520	1	0.19
T9/102	534	1	0.19
MAD20	510	1	0.20
RO33	530	3	0.57
Subclones:			
HB3 sc1	509	29	5.69
HB3 sc2	509	35	6.87
HB3 sc3	517	22	4.16
HB3 sc4	510	19	3.73
HB3 sc5	534	18	3.37
HB3 sc7	306	23	7.51
HB3 sc10	538	5	0.93
HB3 sc11	516	10	1.94

Similarly low proportions of parasites were seen reactive with antibodies to the C-terminal region although fewer parasites were counted (data not shown).
