# Supplementary material for: Population Genomic Scan for Candidate Signatures of Balancing Selection to Guide Antigen Characterization in Malaria Parasites
Source: PLoS Genet. 2012 Nov 1;8(11):e1002992. doi: 10.1371/journal.pgen.1002992 (PMC3486833; doi:10.1371/journal.pgen.1002992)
Supplement: Table S4 — Sequences of primers and probes, and assay conditions used for quantitative real time PCR to assay transcript abundance of each of the six msp3-like genes. (PDF) [file pgen.1002992.s007.pdf]

Supplementary Table S4. Sequences of primers and probes used for quantitative real time PCR to assay transcript abundance of each of the six *msp3*-like genes

| Locus | Gene | Nucleotide Positions | Sequences of primers (F and R) and probes (P) |
|---|---|---|---|
| PF10_0345 | msp3 | 894-926 | F: 5'-GGAAGCACAGAATTTAATTTCTAAAAACCAGAA-3' |
| | | 968-996 | R: 5'-TCCCTTGATTAAACCAGCTAAAGTTTTCA-3' |
| | | 943-962 | P: 5'-CTTTCAGCAGCTTCTTTTAC-3' |
| PF10_0346 | msp6 | 833-866 | F: 5'-AAGAAAAAAAGGAAGAAGAAGAAAAAAAGGAAGA-3' |
| | | 914-944 | R: 5'-TCACTTGGTGAACTATATTTTTGTTCCTCTT-3' |
| | | 885-902 | P: 5'-ACCAGACAATGAAATTAC-3' |
| PF10_0347 | h101 | 1134-1171 | F: 5'-CTATAAGGATAATGATAAATCAGAAAAAACTGCACAAA-3' |
| | | 1203-1236 | R: 5'-TCTTCTTATGGTAGCATCTAATTCATTTTTTCCA-3' |
| | | 1172-1189 | P: 5'-TCAGAGCTGTGATTAATG-3' |
| PF10_0348 | dblmsp | 1537-1568 | F: 5'-GTAACGCAAAGAGGAAATAATAACTACAACAA-3' |
| | | 1635-1676 | R: 5'-TCTTCTTCATCCTTTGATGTTAATTTTATTAATTCTAGAGAA-3' |
| | | 1586-1602 | P: 5'-AAGAGCACCAGAACCCA-3' |
| PF10_0352 | h103 | 941-982 | F: 5'-AATTACAAGAAAATGAAGATGATGAGGATAATGTAAATTTAG-3' |
| | | 1077-1120 | R: 5'-CATGATCTTCTAATGATTTTTTTTCTTCAGTTTTTTTATATTCA-3' |
| | | 1035-1051 | P: 5'-CATCTTGCGTAGAATCT-3' |
| PF10_0355 | mspdbl2 | 12-31 | F: 5’-AGGTAGCGTGAGCGAAGAAT-3’ |
| | | 107-127 | R: 5’-TGTTCAAGAGATGATGACCCA-3’ |
| | | 51-77 | P: 5’-TCCTTCTAAGATTGACGACCGTTTGGA-3’ |
